# Spectroscopic Characterization of Copper-Chitosan Nanoantimicrobials Prepared by Laser Ablation Synthesis in Aqueous Solutions †

**DOI:** 10.3390/nano7010006

**Published:** 2016-12-30

**Authors:** Maria Chiara Sportelli, Annalisa Volpe, Rosaria Anna Picca, Adriana Trapani, Claudio Palazzo, Antonio Ancona, Pietro Mario Lugarà, Giuseppe Trapani, Nicola Cioffi

**Affiliations:** 1 IFN-CNR, Physics Department “M. Merlin”, Bari 70126, Italy; maria.sportelli@uniba.it (M.C.S.); annalisa.volpe@uniba.it (A.V.); pietromario.lugara@uniba.it (P.M.L.); 2 Chemistry Department, Università degli Studi di Bari “Aldo Moro”, Bari 70126, Italy; rosaria.picca@uniba.it; 3 Physics Department, Università degli Studi di Bari “Aldo Moro”, Bari 70126, Italy; 4 Department of Pharmacy-Drug Sciences, Università degli Studi di Bari “Aldo Moro”, Bari 70126, Italy; adriana.trapani@uniba.it (A.T.); claudio.palazzo@uniba.it (C.P.); giuseppe.trapani@uniba.it (G.T.)

**Keywords:** copper nanoparticles, chitosan, laser ablation, X-ray photoelectron spectroscopy, FTIR

## Abstract

Copper-chitosan (Cu-CS) nanoantimicrobials are a novel class of bioactive agents, providing enhanced and synergistic efficiency in the prevention of biocontamination in several application fields, from food packaging to biomedical. Femtosecond laser pulses were here exploited to disrupt a Cu solid target immersed into aqueous acidic solutions containing different CS concentrations. After preparation, Cu-CS colloids were obtained by tuning both Cu/CS molar ratios and laser operating conditions. As prepared Cu-CS colloids were characterized by Fourier transform infrared spectroscopy (FTIR), to study copper complexation with the biopolymer. X-ray photoelectron spectroscopy (XPS) was used to elucidate the nanomaterials’ surface chemical composition and chemical speciation of the most representative elements. Transmission electron microscopy was used to characterize nanocolloids morphology. For all samples, ξ-potential measurements showed highly positive potentials, which could be correlated with the XPS information. The spectroscopic and morphological characterization herein presented outlines the characteristics of a technologically-relevant nanomaterial and provides evidence about the optimal synthesis parameters to produce almost monodisperse and properly-capped Cu nanophases, which combine in the same core-shell structure two renowned antibacterial agents.

## 1. Introduction

Laser ablation synthesis in solution (LASiS) is a modern and intriguing metal nanoparticle production route, which offers several advantages [1]. Firstly, it is clean and straightforward, and it is based on the controlled disruption of a metallic target. It does not require neither reducing agents nor the use of precursors, such as metal salts. Moreover, capping agents are not mandatorily required, since the solvent itself can offer a modest stabilizing effect on as-prepared nanophases [2]. Secondly, as-prepared nanoparticles are highly reactive, and immediately after their preparation, they can be further processed by introducing specific ligands or functionalities via flow reactors [3,4]. Thirdly, several experimental parameters, including laser operating conditions, can be used to tune the features of the produced nanomaterial [5]. Finally, the method has been proven to be mature for the large-scale production of nanomaterials, since nanoparticle productivities of a few grams per hour have been recently demonstrated by using a high-power and high-repetition-rate ultrafast laser source together with a high-speed polygon scanner [6]. In the field of antimicrobial nanomaterials (nanoantimicrobials (NAMs)), LASiS methods have been recently employed to the preparation of technologically-relevant nanocolloids, which have been applied as active additives in food packaging [7,8], biosensing and diagnostics [4,9], catalysis [10], optoelectronics [11], and so on. Chitosan (CS), or poly-β-(1-4)-2-amino-2-deoxy-d-glucose, is a poly-aminosaccharidic cationic polymer, produced by the *N*-deacetylation of chitin, which is frequently used as an antibacterial agent, as well [12,13,14,15,16,17]. After cellulose, CS is the second most abundant polysaccharide on Earth; it is inexpensive and biocompatible [18]. The attractive properties of CS and its derivatives have initiated numerous research activities, resulting in an ever-increasing amount of new potential applications [19,20,21,22]. Noteworthy, CS can efficiently chelate and/or coordinate metals, ensuring the possibility to prepare metal-containing coordination polymers and composites [23]. In particular, metal-chitosan (Me-CS) composites have many fascinating applications in catalysis, biosensors and biomaterials [24,25]. A number of studies have been issued on CS combination with different inorganic nanostructures, such as Pd [26,27], Au [28,29,30,31,32], Ag and its compounds [33,34,35,36,37,38,39,40], Pt [27,41], Fe and its oxides [42,43,44], ZnO [20,45,46], zeolite [47], silica [48] and graphene [49], just to cite a few. The inclusion of copper nanophases in CS matrix is quite diffused, as well [50,51,52,53,54,55,56,57]. The literature on copper-chitosan (Cu-CS) composites mostly deals with chemically-synthetized materials, obtained by the reduction of copper salts/precursors in aqueous [50,52,53,55,58] or organic media [54,56]. Since copper nanoparticles (CuNPs) readily oxidize under ambient conditions, most of the reported synthetic methods adopt CS as the stabilizing agent, added in situ during the colloid preparation. Less frequent is the use of CS as a post-synthesis protecting agent for colloid stabilization [51]. Cu-CS composites are widely used in catalysis and biocatalysis [59,60,61,62,63]. CS modified with Cu(II) ions has been used as a convenient substrate for enzyme immobilization [60], with large thermal and operational stability. Furthermore, visible light-activated Cu/Cu_2_O-CS nanocomposites are effective and inexpensive photo-catalysts used in the degradation of organic dyes [59]. Implemented in filtration systems, these composites are useful tools for the purification of wastewater, based on the massive elimination of alcohols and phenols from effluents [61]. Cu-CS composites are also able to bind, by electrostatic interaction, DNA chains and to induce DNA catalytic cleavage [63]. In the framework of our activities in the field of nanoantimicrobials, we recently published a letter proposing a one-step approach for the direct LASiS synthesis of Cu-CS composites in an aqueous CS solution [8]. The importance of developing Cu-CS combinations, resides in their perspective application as nanoantimicrobial agents [64,65], synergistically coupling the well-known bioactivity of both phases. Cu-Cs composites have been already synthesized exploiting chemical methods, i.e., copper salt reduction in the presence of CS, the latter acting as both a reducer and a stabilizer. Anyhow, such methods generally require the use of potentially toxic and/or harmful chemicals [66] (NaBH_4_, as an example) or may lead to the formation of an NP powder [52,67,68]. The LASiS method provides, instead, stable colloids without the use of reducing agents or potentially dangerous substances. Regarding antimicrobial applications and considering the preparation method herein studied, it is noteworthy that Zamiri et al. proposed an LASiS route to silver-chitosan (Ag-CS) composites for biomedical applications [69], and we are presently exploring LASiS routes to multi-metal nanoantimicrobials [70]. In fact, silver and copper nano-antimicrobials exert their bioactivity by means of different mechanisms, and even considering the common aspects, they provide different releases of bioactive ions in the most diffused contact media, such as physiological solution, sweat, food matrices, etc. [64]. Due to the lower cost and the easier bioactivity mechanism, based almost exclusively on the ionic release aspects [71,72], copper-based nanoantimicrobials are well suited for food packaging applications. In this study, for the first time, we report on the detailed analytical characterization of Cu-CS nanomaterials synthetized via the LASiS route in aqueous media. The organic phase content was optimized in order to obtain the best nanomaterial morphology, and spectroscopic results were used to define its surface and bulk chemical composition. X-ray photoelectron spectroscopy (XPS), transmission electron microscopy (TEM), ξ-potential measurements and Fourier transform infrared (FTIR) spectroscopy techniques were used to study materials’ physicochemical properties, thus supporting their application as antimicrobial additives in food packaging and biomedical fields.

## 2. Results

### 2.1. Synthesis of Cu-CS Nanocomposites

In this work, CuNPs were obtained by femtosecond-pulsed laser ablation of a Cu solid target immersed in HAc solutions containing CS at different concentrations (refer to Section 3.2 for further details). As already observed [8], the CS content influenced the efficiency of NP production, and the amount of the ablated mass progressively decreased with increasing CS concentration. This evidence was related to the attenuation of the incident laser beam for more concentrated solutions. The extent of these phenomena was quantified by irradiating CS solutions at different concentrations and measuring the transmitted light intensity through an optical path equivalent to the liquid thickness above the metal target. Thus, the highest attenuation of the laser radiation was found for the samples with the highest CS content, in quantitative agreement with the decrease of the ablated CuNPs mass. The investigation of the physical mechanism underlying the laser beam attenuation at higher CS concentration was beyond the scope of the present paper.

### 2.2. Morphological Characterization

**Figure 1:**
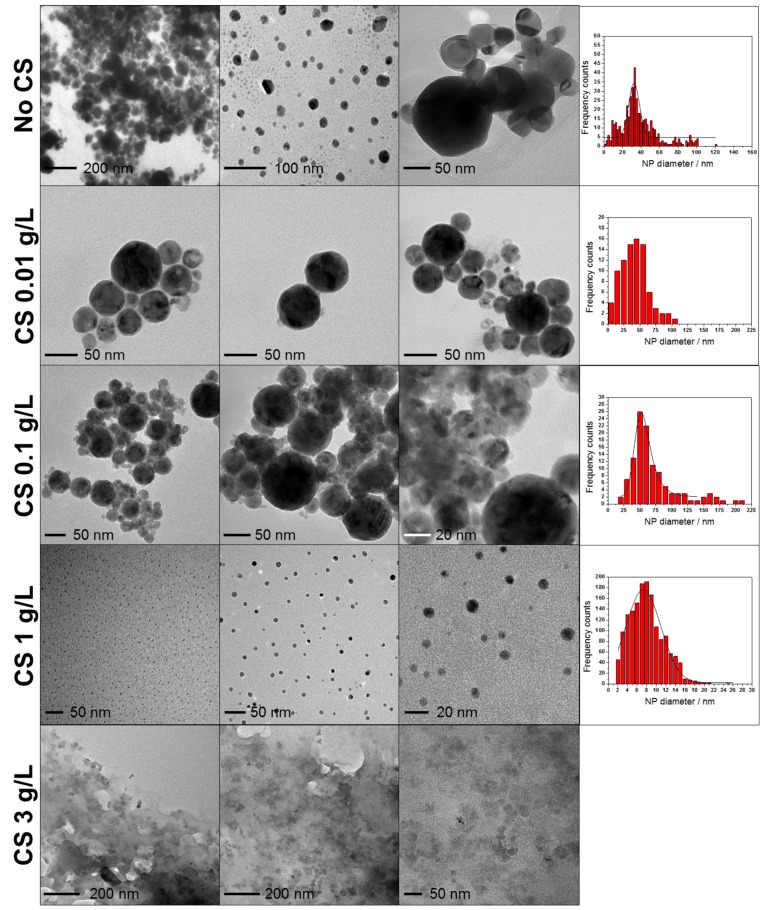
Transmission electron microscopy (TEM) images of laser-generated Cu nanoparticles (CuNPs) in the presence of different chitosan (CS) concentrations and corresponding size distribution histograms. Sizing could not be performed on 3 g/L CuNPs-CS nanocomposite, because of the massive presence of organic matrix.

The morphological characterization of laser-generated CuNPs was carried out by TEM. Typical images of CuNPs obtained by laser ablation at different CS concentrations are reported in Figure 1, along with corresponding size-distribution histograms. In absence of CS (first row in Figure 1), CuNPs appeared extremely polydisperse, with extensive aggregation. Large NP size and significant polydispersity were also evidenced for Cu-CS NPs prepared in absence or at low CS concentrations (e.g., 0.01 and 0.1 g/L), while the best (ultrafine and monodisperse) CuNP size distribution, centered at 7 ± 4 nm, was obtained at a CS concentration of 1 g/L. A further increase of polymer concentration resulted in a massive encapsulation of CuNPs into the dispersing matrix, leading to thick and irregular composite films, which made CuNP visualization and sizing almost impossible.

### 2.3. XPS Characterization

XPS was used to elucidate the surface chemical composition of CS-capped CuNPs. Results from surface elemental analyses are reported in Figure S1, as a function of the CS concentration. Copper, carbon, nitrogen and oxygen were found on the surface of all of the composite nanomaterials. The presence of nitrogen could be exclusively attributed to the CS amino groups. Copper surface percentage decreased as the polymer concentration was raised; consequently, an increment in carbon, oxygen and nitrogen was observed.

**Figure 2:**
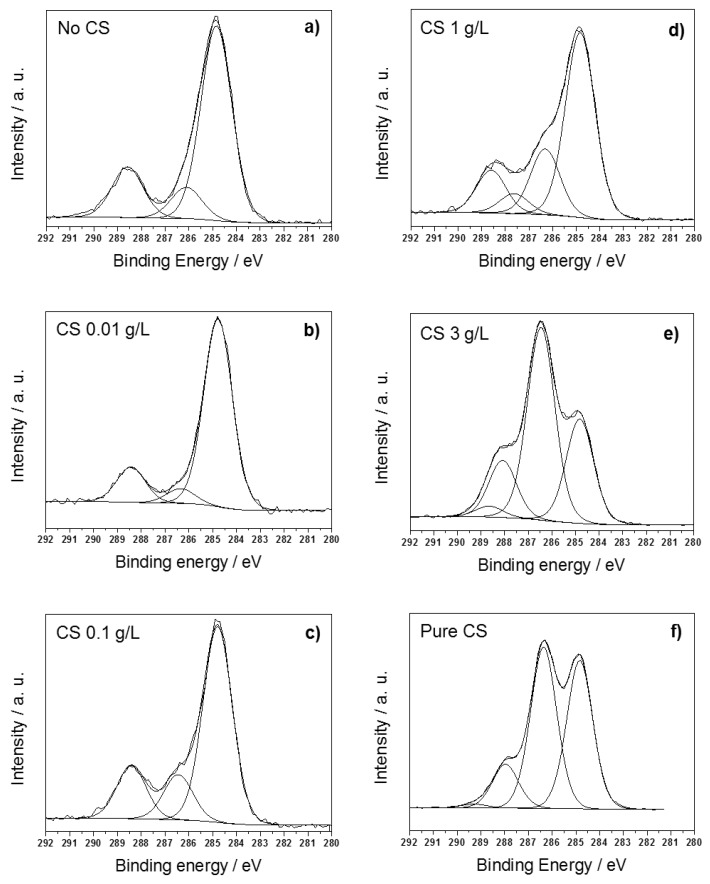
C1s high-resolution regions of freshly-prepared CuNPs-CS nanocomposites synthetized at a CS concentration of: (**a**) no CS; (**b**) 0.01 g/L; (**c**) 0.1 g/L; (**d**) 1 g/L; (**e**) 3 g/L; (**f**) pure CS.

C1s high-resolution spectra for all samples are reported in Figure 2. It is evident how the CS concentration dramatically influenced the line shape of C1s signals. At low CS concentrations, e.g., 0.01 and 0.1 g/L, corresponding to a metal/biopolymer mass ratio comprised between ~50 and five, three components were found. Moving from low to high binding energy (BE), the first peak, falling at BE = 284.8 ± 0.1 eV, was attributed to aliphatic carbon; the second one, falling at BE = 286.4 ± 0.2 eV, was attributed to –C–OH and –C–N groups; the third one, centered at 288.4 ± 0.2 eV, was attributed to the –COOH chemical environment [73]. In these circumstances, carbon surface speciation resembled that of adventitious contaminants (Figure 2a), rather than the one of pure CS (Figure 2f).

In fact, at CS concentrations ≥ 1 g/L, the C1s line shape changed significantly, and a fourth component appeared at BE = 287.8 ± 0.2 eV, ascribable to O–C–O moieties typical of the CS matrix. The position of the –COOH component also changed and raised up to 288.7 ± 0.2 eV, which is consistent with the prevalence of chitosan in the organic substance on the NP surface.

**Figure 3:**
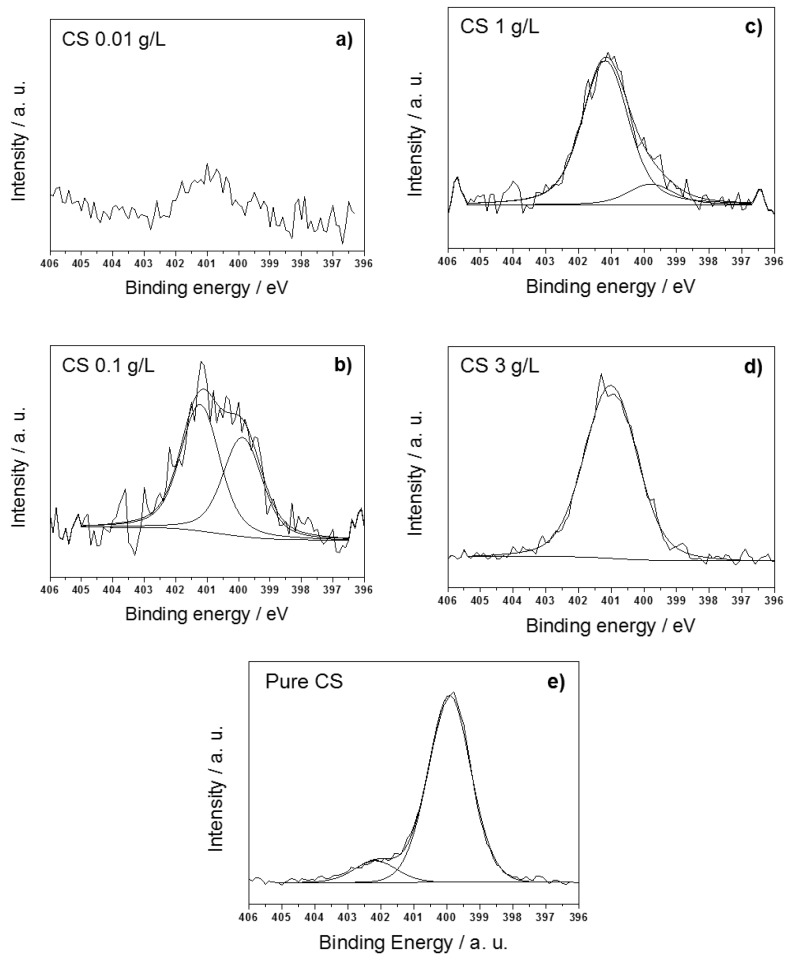
N1s high-resolution regions of freshly-prepared CuNPs-CS nanocomposites synthetized at CS concentration of: (**a**) 0.01 g/L; (**b**) 0.1 g/L; (**c**) 1 g/L; (**d**) 3 g/L; (**e**) pure CS.

**Figure 4:**
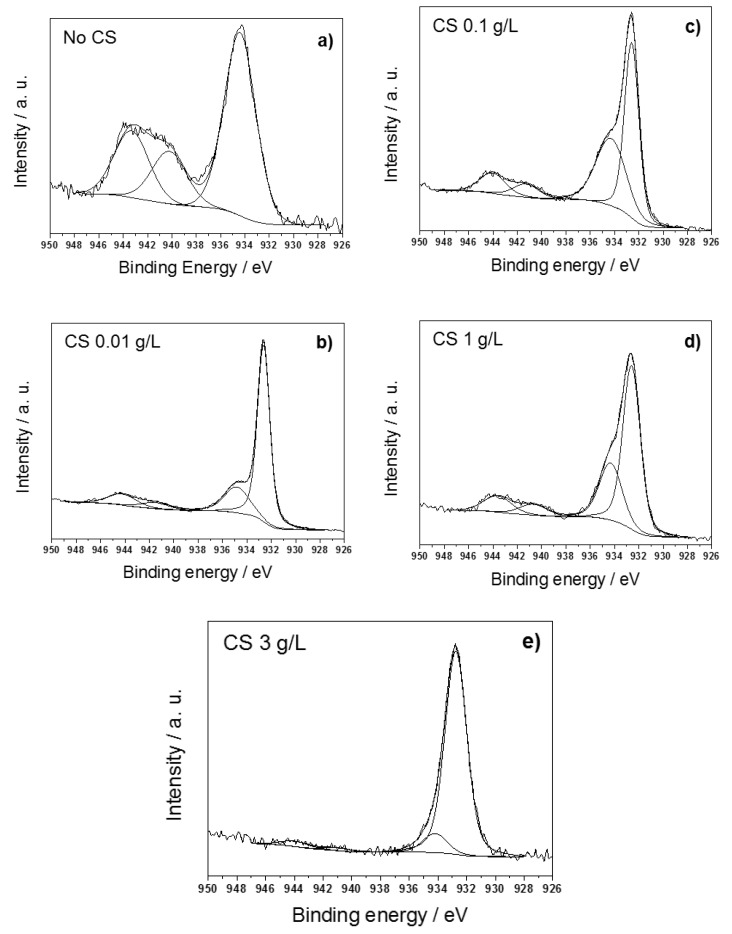
Cu2p_3/2_ high-resolution regions of freshly-prepared CuNPs-CS nanocomposites synthetized at CS concentrations of: (**a**) no CS; (**b**) 0.01 g/L; (**c**) 0.1 g/L; (**d**) 1 g/L; (**e**) 3 g/L.

Finally, composites obtained at the highest CS loading (3 g/L, corresponding to a Cu/CS mass ratio of about 0.1) showed the a C1s spectrum expected for and very similar to that of pure CS [74]. N1s spectra at all CS concentrations are reported in Figure 3, along with the one related to pure CS., for comparison. At the lowest CS loading, this element was not quantifiable, and its X-ray photoelectron (XP) main signal could not be subjected to curve-fitting. In all of the other cases, two components were observed: the one at lower BE values (399.9 ± 0.2 eV) was related to the amino groups from the CS matrix; the second one (401.3 ± 0.2 eV) to protonated –N–H^+^ moieties [73]. Positions of both nitrogen components were in agreement with those reported for pure CS [75], within the experimental error. The ammonium chemical environment was the most abundant nitrogen species at all biopolymer concentrations, and this is reasonably due to the acidic aqueous solution, protonating CS amino groups. O1s high-resolution regions (Figure S2) were curve-fitted using two or three components. In absence of stabilizer and at low CS loadings, e.g., when CS probably was not enough to stabilize Cu, a component related to O–Cu, from cuprous and cupric oxides, was evident at 530.5 ± 0.2 eV [76]. The other two signals were attributed to the O=C (531.7 ± 0.2 eV for CS ≤ 0.1 g/L, 531.4 ± 0.2 eV for CS ≥ 1 g/L), and HO–C (532.9 ± 0.2 eV) chemical environments [73]. Differences in BE of the O=C chemical environment at high CS concentrations could be easily correlated with the significant change in C1s line shapes and were consistent with a progressive prevalence of the biopolymer chemical environments. Cu2p_3/2_ high-resolution regions (Figure 4) showed the presence of two Cu chemical environments. The first one, centered at 932.7 ± 0.2 eV, was ascribable to Cu(0)/Cu(I) moieties [73]. The second one, at about 934.4 ± 0.3 eV, was attributed to Cu(II) species. Its presence was confirmed by the two intense shake-up features falling between 939 eV and 946 eV [77]. Even though XPS analyses were carefully performed on freshly-prepared samples, minimizing their air exposure, copper speciation outlined the presence of Cu(II) species in all samples. At low CS concentrations (≤1 g/L), the degree of NP surface oxidation into CuO was high, as shown by the reported spectra. No Cu(0) was detected on CuNPs prepared in the absence of CS instead, thus confirming the good stabilizing properties of this biopolymer. In our previous work, colloid aging resulted in an almost complete oxidation of Cu moieties [8]. In this study, the effect of air exposure was also investigated as a function of the CS concentration, by analyzing Cu-CS nanoparticles after one month from their preparation. Aging in air resulted in an extensive oxidation of Cu particles at CS loadings <3g/L, as outlined in [8].

### 2.4. FTIR Characterization and ξ-Potential Measurements

**Figure 5:**
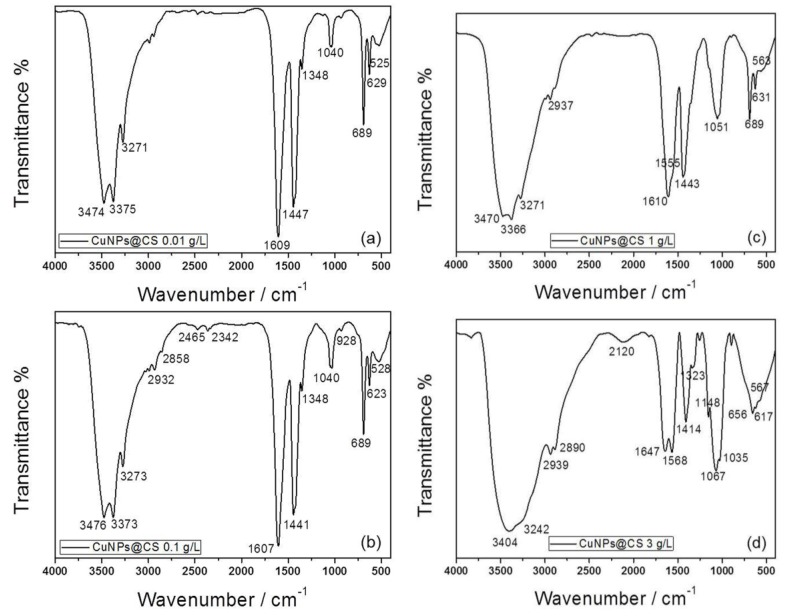
Fourier transform infrared (FTIR) spectra of freshly-prepared CuNPs stabilized by CS (CuNPs@CS nanocomposites, “@” stands for “stabilized by”), synthetized at CS concentrations of: (**a**) 0.01 g/L; (**b**) 0.1 g/L; (**c**) 1 g/L; (**d**) 3 g/L.

FTIR spectra of Cu-CS samples are reported in Figure 5. It is evident how CS concentration dramatically influenced peak shapes and intensities. In fact, while the spectrum of CuNPs-CS composite with a polymer concentration of 3 g/L (Figure 5d) was consistent with the one already reported for pure CS (Figure S3) [78], those related to lower CS loadings (Figure 5a–c) showed significant differences. Bands falling at 3480–3270 cm^−1^, associated with the N–H and O–H stretching modes of moieties involved in hydrogen bonds [79], exhibited a lower broadening at low CS loadings. This is attributed to a decrease in hydrogen bonding induced by the interaction of these moieties with copper [80]. Indeed, it is known in the literature that Cu complexation to CS occurs by means of amino and carbonyl groups [81]. The occurrence of complexation was confirmed by the trend shown by the peak at 1570 cm^−1^, which is attributed to γ-(NH_2_) bending [78]. It was quite intense and defined in the case of Cu-CS composites with a polymer concentration of 3 g/L and in the case of pure CS [78] (Figure S3). This band was scarcely appreciable with a CS concentration of 1 g/L (Figure 5c), and it disappeared completely at lower loadings. As reported in the literature, the intensity of this band decreases, increasing the copper complexation degree [82]. This could be indicative of a saturation of the biopolymer complexation sites [78].

The involvement of carbonyl functional groups in the complexation of Cu to the polymer backbone was also hypothesized [81]. C–O stretching was detectable through an intense band at about 1060–1040 cm^−1^ in bare CS (Figure S3), but its intensity decreased dramatically as the complexation sites were filled, as shown in Figure 5. Lastly, the entire spectral region between 690 cm^−1^ and 520 cm^−1^ was due to Cu–N and Cu–O stretching. As expected, these bands were absent in pristine CS, and their intensity was proportional to Cu concentration in the colloid. In fact, they were particularly intense for lower loadings, which provided higher ablation yields. Finally, it is worth noting that all spectra were recorded using freeze-dried samples, implying that the Cu-CS complexes remained stable after solvent evaporation and that the polymeric structure was not altered by the freeze-drying process. We checked for possible artifacts related to this issue, and we compared the FTIR spectra of pure CS analyzed as such or obtained by freeze-drying of a concentrated CS solution in 0.1 *v*/*v* % HAc. These data (reported in Figure S3) showed how the sudden solvent evaporation was responsible for the reduced presence of hydrogen bonding and for the partial degradation of amide functionalities. This was confirmed by a sharper band at ~3400 cm^−1^ and by a weak feature between 1070 cm^−1^ and 1030 cm^−1^ for the lyophilized CS, if compared with the bare one. ξ-potential measurements on Cu-CS nanocolloids obtained at different CS loadings always returned positive values, which are in agreement with XP N1s spectra, outlining a massive presence of positively-charged ammonium moieties in the CS shell stabilizing the inorganic nanophases. Although ξ-potential measurements presented a certain batch-to-batch variability, in Figure S4, it can be observed that samples obtained at the highest CS concentration generally showed a slightly lower potential. To account for this finding, we hypothesized that these nanomaterials are characterized by a very different structure, as compared to other samples. In fact, TEM images of Figure 1 show the presence of a thin CS shell surrounding the Cu cores for samples obtained at CS ≤ 1 g/L, while for the highest loading, copper cores appear fully immersed in a thick organic matrix (visible as a low-contrast background area), which might surround the primary particles and mask/reduce their surface potential.

## 3. Materials and Methods

### 3.1. Materials

Copper targets (diameter 10 mm, 1 mm thick, 99.999%) were purchased from GoodFellow Ltd, (Huntingdon, UK). Aluminum oxide (>95%), used for the mechanical polishing of Cu targets, chitosan (medium molecular weight, 190–310 kDa, 75%–85% deacetylated according to manufacturing instructions) and acetic acid (HAc, glacial, ≥99.85%) were purchased from Sigma Aldrich (Milan, Italy). Milli-Q water was used throughout the experiments. All chemicals were of analytical grade and used as received.

### 3.2. LASiS Synthesis of Nanomaterials

A Cu target was mechanically polished with wet SiC grinding paper and cleaned in water with an ultrasonic bath. Then, it was placed on the bottom of a beaker filled with 10 mL of a CS solution at four different concentrations (i.e., 0.01, 0.1, 1.0 and 3.0 g/L), prepared in 0.1 *v*/*v* % HAc. Laser ablation was carried out at room temperature and atmospheric pressure by irradiating the immersed Cu target with a focused laser beam. To prepare Cu-CS composites, a femtosecond laser system (Sci-series, Active Fiber Systems GmbH, Jena, Germany) was used, delivering 650-femtosecond laser pulses at the fundamental wavelength of 1030 nm (see Figure S5).

This laser source is able to operate at a maximum pulse energy of 100 μJ, with repetition rates from 50 kHz to 500 kHz or at a maximum average power of 50 W for higher repetition rates, up to 10 MHz. Cu-CS composites were generated by operating the laser at a repetition rate of 100 kHz and 70 μJ of pulse energy, corresponding to 7 W of average power. The laser beam was coupled into a galvo-scan-head (HurrySCAN II, ScanLab, Jena, Germany), having an entrance aperture of 14 mm and equipped with dielectric mirrors with antireflection (AR) coating at the laser wavelength. The beam was then focused through a 100-mm focal-length F-Theta lens onto the copper target surface, located about 5 mm beneath the liquid surface. During the laser ablation process, the laser beam was scanned over the entire copper disc surface through concentric circular patterns with an interline distance of 100 μm. A number of 800 consecutive full patterns was performed at a scan speed of 300 mm/s. The mass of as-synthesized nanoparticles was determined by differential weighting of the copper target before and after the ablation process. The mass of copper dispersed as nanoparticles ranged from 5.1 ± 0.2 mg (value relevant to a CS concentration of 0.01 g/L) to 3.2 ± 0.6 mg (at a CS loading of 3 g/L).

### 3.3. Morphological and Spectroscopic Characterization

TEM microscopy was performed with an FEI Tecnai 12 instrument (FEI Italia SRL, Milan, Italy) operated at 120 kV (filament: LaB_6_), by dropping the colloidal samples on a Formvar^®^-coated Cu grid (300 mesh, Agar Scientific, London, UK). The microscope was calibrated by using the S106 Cross Grating (2160 lines/mm, 3.05 mm) supplied by Agar Scientific. Cu-CS composites (100 μL) were drop-cast on Pt foils for carrying out XPS characterization using a Thermo Fisher Scientific Theta Probe Spectrometer (Waltham, MA, USA), equipped with a monochromatized Al Kα source (beam spot diameter of 300 μm). All XPS measurements were performed in constant analyzer energy (CAE) mode. Survey and high-resolution spectra (C1s, O1s, Cu2p, N1s and Na1s) were respectively acquired at a pass energy of 150 and 100 eV and with a step size of 1.0 and 0.1 eV. The acquisition time of the entire set of spectra was about 20 min, as an acceptable compromise between satisfying signal-to-noise ratios and minimal exposure time to radiation. We assessed by comparison of C1s and Cu2p spectra acquired at different times during the analysis that changes of the line shape within the analysis time were negligible. Detailed spectra processing was performed by commercial Thermo Avantage^®^ software (v. 5.937, 2014, Thermo Fisher Scientific). Accurate curve-fitting analysis was applied to high-resolution spectra. Surface atomic percentages were determied considering the integrated peak areas and the respective sensitivity factors. The same peak line shape (Gaussian/Lorentzian ratio and full width at half maximum) parameters were employed for the curve fitting of components belonging to the same high-resolution spectrum. XPS peak intensities were obtained after a Shirley background removal, using Scofield sensitivity factors. Spectra were corrected for charge compensation effects by offsetting the binding energy relative to the aliphatic component of the C1s spectrum, which was set to 284.8 eV. Differently from what was previously reported [8], samples were here analyzed immediately after their synthesis, in order to limit hydrocarbon contamination and NP surface oxidation induced by air. In fact, air exposure can affect Cu chemical speciation, promoting extensive nanoparticle oxidation. In order to better argue this point, XPS analyses were repeated on the same samples after one month from their preparation. Fourier transform infrared (FTIR) spectroscopy measurements were performed on freeze-dried samples, on a Bio-Rad FTS6000 instrument (Hercules, CA, USA) with a DTGS detector in transmittance mode (resolution 2 cm^−1^, range 4000–400 cm^−1^) and processed using the BIORAD WinIR^®^ Pro software. Samples were prepared as KBr (Sigma Aldrich, Milan, Italy, FTIR grade, ≥99% trace metals basis) pellets, grinding a proper quantity of powder in an agate mortar. The determination of ξ-potentials was performed using laser Doppler anemometry (Zetasizer NanoZS, ZEN 3600, Malvern, UK) filling the entire cuvette with the sample dispersion.

## 4. Conclusions

In this work, copper nanoparticles generated by LASiS in chitosan aqueous solutions were characterized by TEM, XPS, FTIR and ξ-potential measurements, in order to assess their morphological and chemical properties. Increasing amounts of CS in the synthesis environment led to a better NP stabilization (towards NP aggregation and precipitation). On the other hand, they also reduced metal surface availability, as shown by XPS elemental analyses. CS-stabilized CuNPs with an exceptional size homogeneity were obtained with a CS loading of 1 g/L. Partial surface oxidation of as-prepared NPs was always observed by XPS. However, NP oxidation is not a problem for their further applications in antimicrobial Cu-CS composites. In fact, the role of cupric oxide as intermediate species, affording a controlled ion release and the consequent bioactivity, has been already demonstrated in several studies [64,83,84]. FTIR analyses indicate that CuNPs are involved in complexation phenomena with CS amino and carboxylate functional groups. The nanocomposites herein characterized are attractive bioactive materials, possessing good antibacterial activity, as demonstrated by preliminary biological tests performed with *Escherichia coli* [8]. Finally, LASiS-prepared copper nanocolloids offer interesting prospects, since they can be easily mixed into solutions of commercial polymers, such as poly-lactic acid or Eudragit^®^, paving the way for the preparation of composites to be used in food packaging or biomedical fields [85].

## Supplementary Materials

The following items are available online at http://www.mdpi.com/2079-4991/7/1/6/s1.
