# Supplementary material for: Spectroscopic Characterization of Copper-Chitosan Nanoantimicrobials Prepared by Laser Ablation Synthesis in Aqueous Solutions
Source: Nanomaterials (Basel). 2016 Dec 30;7(1):6. doi: 10.3390/nano7010006 (PMC5295196; doi:10.3390/nano7010006)
Supplement: Supplementary file 1 [file nanomaterials-07-00006-s001.pdf]

Supplementary Materials: Spectroscopic Characterization of Copper-Chitosan Nanoantimicrobials Prepared by Laser Ablation Synthesis in Aqueous Solutions

Maria Chiara Sportelli, Annalisa Volpe, Rosaria Anna Picca, Adriana Trapani, Claudio Palazzo, Antonio Ancona, Pietro Mario Lugarà, Giuseppe Trapani and Nicola Cioffi

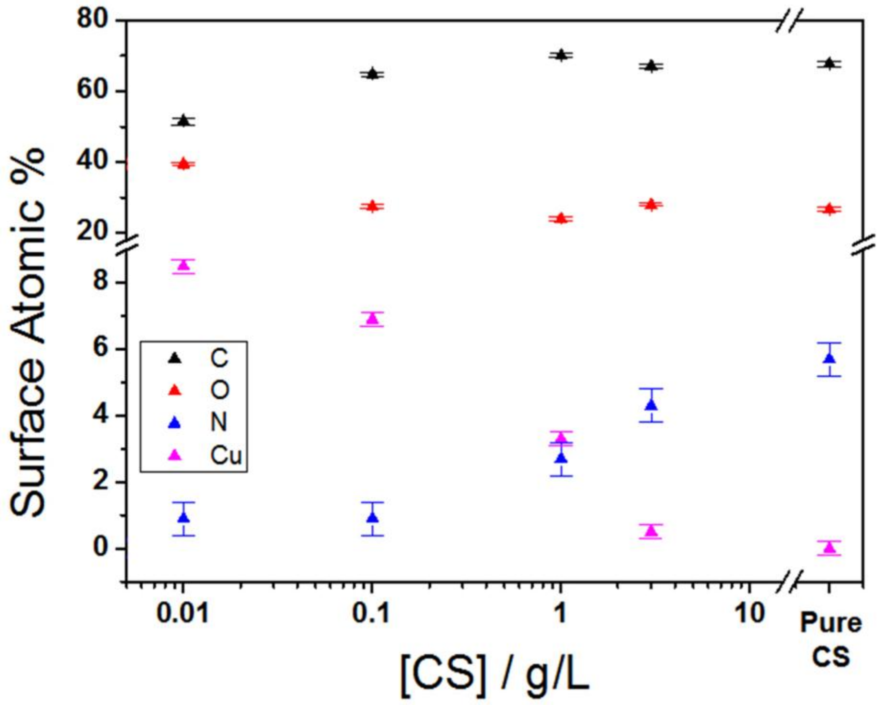

Figure S1. C, O, Cu and N surface atomic percentages as a function of CS loading. Data about pure CS are also reported, for comparison.

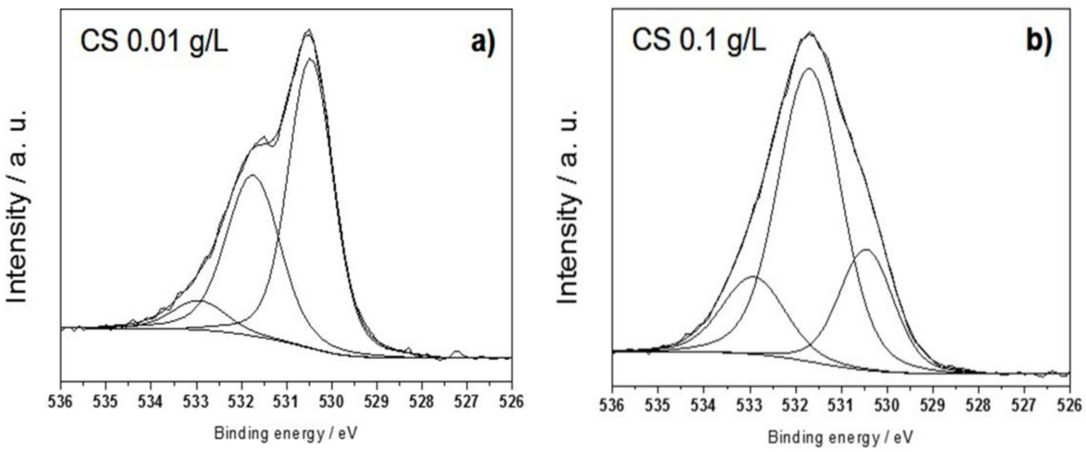

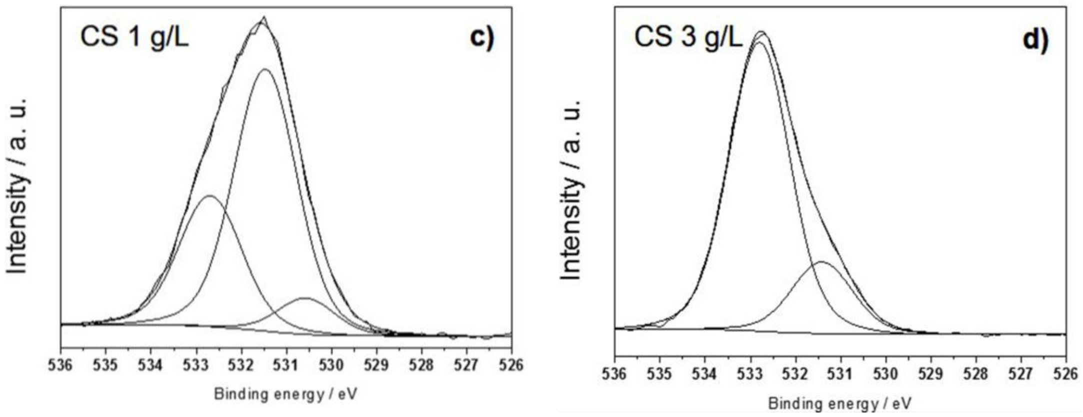

Figure S2. O1s high-resolution regions of freshly-prepared CuNPs-CS nanocomposites synthetized at CS concentrations of: (**a**) 0.01 g/L; (**b**) 0.1 g/L; (**c**) 1 g/L; (**d**) 3 g/L.

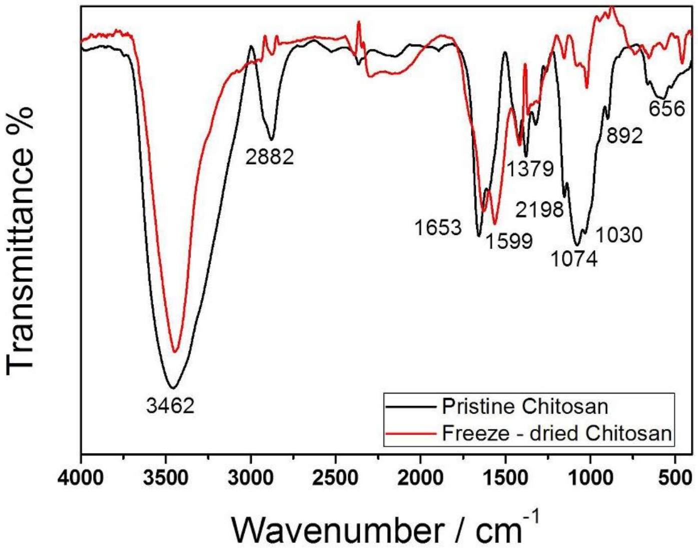

Figure S3. Comparison between FTIR spectra relevant to pristine (black line) and freeze-dried (red line) chitosan samples.

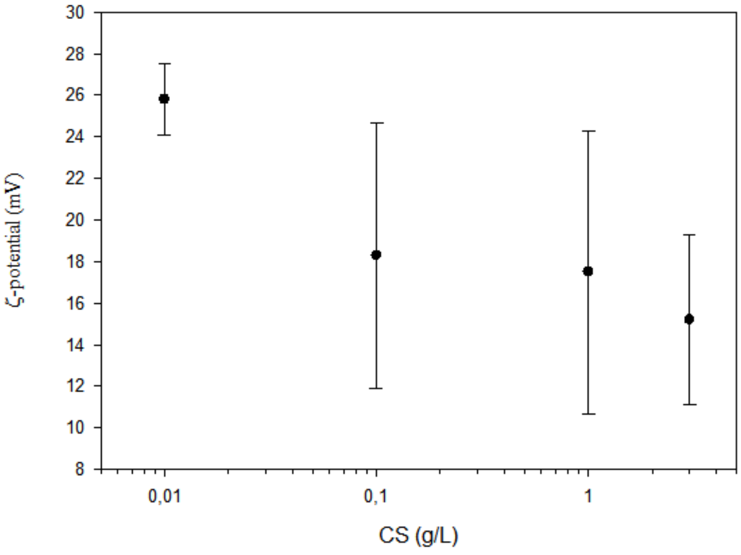

Figure S4. Nanoparticle ζ-potential values as a function of CS concentration.

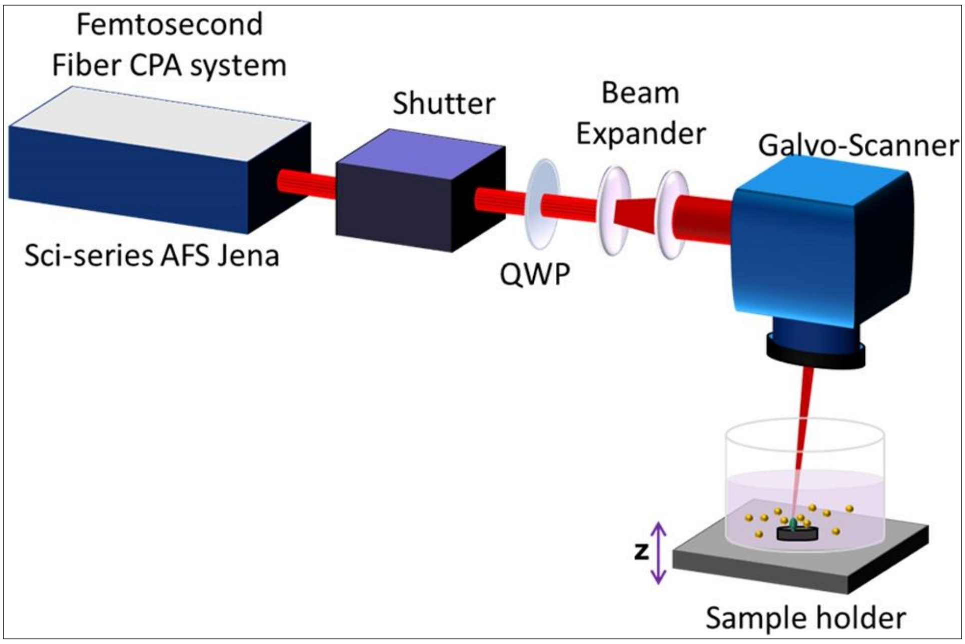

Figure S5. Schematic representation of the laser ablation synthesis in solution (LASiS) experimental setup. CPA = chirped pulse amplification; AFS = Active Fiber Systems; QWP = quarter-wave-plate.

© 2016 by the authors. Submitted for possible open access publication under the terms and conditions of the Creative Commons Attribution (CC-BY) license (http://creativecommons.org/licenses/by/4.0/).
